# Relevance of Serum Levels and Functional Genetic Variants in Vitamin D Receptor Gene among Saudi Women with Gestational Diabetes Mellitus

**DOI:** 10.3390/nu15194288

**Published:** 2023-10-08

**Authors:** Imran Ali Khan, Maysoon Abdulhadi Alhaizan, Salwa Mohamed Neyazi, Malak Mohammed Al-Hakeem, Amal F. Alshammary

**Affiliations:** 1 Department of Clinical Laboratory Sciences, College of Applied Medical Sciences, King Saud University, Riyadh 11433, Saudi Arabia; aalshammary@ksu.edu.sa; 2 Department of Obstetrics and Gynecology, College of Medicine, King Khalid University Hospital, King Saud University, Riyadh 11451, Saudi Arabia; malhaizan@ksu.edu.sa (M.A.A.); sneyazi@ksu.edu.sa (S.M.N.); malhakeem@ksu.edu.sa (M.M.A.-H.)

**Keywords:** vitamin D or 25-hydroxyvitamin D (25(OH)D), VDR gene, ApaI, BsmI, FokI, TaqI, GDM and non-GDM women

## Abstract

*Background*: This study explored the association between ApaI–TaqI Single Nucleotide Polymorphisms (SNPs) in a Vitamin D receptor (VDR) and the risk of Gestational Diabetes Mellitus (GDM) in Saudi women, along with the serum levels of vitamin D. *Methods*: Ninety women with GDM and 90 non-GDM women were enrolled, based on the inclusion and exclusion criteria for pregnant women enrolled in a single-center study. Blood samples were retrieved from 180 pregnant women using ethylenediaminetetraacetic acid (EDTA) tubes. Serum samples were used to measure the vitamin D, 25-hydroxyvitamin D (25(OH)D or calcidiol), and lipid profiles. Blood was used to measure the hemoglobin A1c levels and to isolate the DNA. The polymerase chain reaction (PCR) was performed for the ApaI (rs79785232), BsmI (rs1544410), FokI (rs2228570), and TaqI (rs731236) SNPs in the *VDR* gene using restriction fragment length polymorphism analysis. Validation was performed using Sanger sequencing. Statistical analyses were performed between the patients with and without GDM using various statistical software packages. *Results*: The Hardy–Weinberg equilibrium analysis was statistically significant (*p* > 0.05). The ApaI, BsmI, and TaqI SNPs were associated with alleles, genotypes, and different genetic models (*p* < 0.05). Vitamin D levels were associated with deficient levels (*p* = 0.0002), as well as with a normal and overweight body mass index (*p* = 0.0004). When vitamin D levels were measured with GDM covariates, the fasting plasma glucose (FPG) (*p* = 0.0001), postprandial blood glucose (PPBG) (*p* < 0.0001), oral glucose tolerance test (OGTT)-1 h (*p* = 0.005), high-density lipoprotein (*p* = 0.022), and low-density lipoprotein cholesterol (LDLc) (*p* = 0.001) levels were significantly different. When similar vitamin D levels were measured for each genotype, we confirmed that the ApaI SNP was associated with sufficient levels (*p* < 0.0001), whereas the BsmI, FokI, and TaqI (*p* < 0.05) were associated with insufficient levels. The logistic regression model confirmed that the first hour of the OGTT (*p* = 0.005) was strongly associated with GDM, whereas the analysis of variance confirmed that FPG and PPBG (*p* < 0.05) were strongly associated with all the SNPs evaluated in the *VDR* gene. Additionally, the second hour of the OGTT (*p* = 0.048) and LDLc (*p* = 0.049) were associated with the ApaI and FokI SNP. Moreover, the first hour OGTT (*p* = 0.045) and lipid profile parameters (*p* < 0.05) were associated. Haplotype analysis revealed positive associations among the examined SNPs, which seemed compatible with the hypothesis that variants and combinations of multiple SNP genotypes enhance the risk of GDM in women. Haplotype analysis revealed that different combinations of alleles, such as AGCC, CATT, CGTC, AGTC, and CATT (*p* < 0.05), were strongly associated. The linkage disequilibrium (LD) analysis showed a strong association with all combinations (*p* < 0.05). Among the gene–gene interactions, all possible combinations showed a positive association (*p* < 0.05). *Conclusions*: Low vitamin D levels were observed in women with GDM. The ApaI, BsmI, and TaqI SNPs were associated with genotype and allele frequencies (*p* < 0.05). Vitamin D and the SNPs in the *VDR* gene were associated, according to the ANOVA, logistic regression, haplotype analysis, LD analysis, and the generalized multifactor dimensionality reduction model (*p* < 0.05).

## 1. Introduction

Diabetes mellitus (DM) is a metabolic disorder resulting from impaired insulin secretion, or a combination of insulin resistance and inadequate insulin secretion [1]. Prediabetes occurs with impaired fasting glucose (IFG), impaired glucose tolerance (IGT), and glycated hemoglobin (HbA1c) at 5.7–6.4% [2]. Based on the American Diabetes Association (ADA) criteria, DM is categorized into: (i) type 1 (T1DM), (ii) type 2 (T2DM), (iii) gestational (GDM), and (iv) other forms including monogenic diabetes syndrome, which is subcategorized into neonatal and maturity-onset diabetes of the young [3]. Women who initially develop diabetes during pregnancy, due to carbohydrate intolerance with no established DM diagnosis, are diagnosed with GDM. The pathogenesis of GDM includes pancreatic β-cell failure caused by placental and genetic factors [4]. Advanced maternal age, elevated weight gain during pregnancy, metabolic inflammation, nutritional deficiency, increased adipose tissue, physical inactivity, oxidative stress, polycystic ovary syndrome (PCOS), the infant born weighing 4 kg plus, a family history of T2DM, and GDM are the major common risk factors for developing GDM during pregnancy [5]. As one of the long-term risks involved, GDM women may be predisposed to developing pancreatic cancer in the future. Women with a history of GDM have a seven-fold increased risk of pancreatic cancer [6]. Additionally, women with GDM have an increased risk of cesarean section delivery, renal disease, preeclampsia, cardiovascular diseases (CVD), and metabolic syndrome (MetS) [7]. Neonates born to women with GDM may develop obesity, fetal macrosomia, congenital malformations, and a high fat mass as an infant [8]. Placentas from GDM pregnancies are larger and heavier than those from normal pregnancies; the thickness of the core part of the placenta and the number of placenta cotyledons are also greater in GDM women. This is affected by the elevated HbA1c levels in maternal hyperglycemia. Furthermore, these changes may cause chronic hypoxemic status and oxidative stress in the placentas of women with GDM, which, in turn, may cause the placenta to undergo neoangiogenesis and hypervascularization through the regulation of hormones like EPO, FGF2, leptin, IGF2, and IL-6, and TNF-α as inflammatory cytokines [9]. In humans, parathyroid hormone-related protein (PTH-rP) and parathyroid hormone (PTH)/PTH-rP receptors are generated by the uterus, placenta, fetal membranes (amnion and chorion), and the developing fetus during pregnancy. By stimulating the placental calcium transport, vasodilating the uteroplacental vasculature, and regulating cellular growth and differentiation, PTH-rP plays a crucial role in fetal growth and development. PTH-rP and corrected calcium levels are considerably greater in pregnant women compared to nonpregnant women, although the median PTH serum concentrations are statistically lower in early pregnancy compared to late pregnancy. PTH-rP promotes insulin expression in pancreatic β-cells, and it has been shown that T2DM patients have greater blood PTH-rP levels than control patients [10].

Currently, the global prevalence of GDM is approximately 13–26% [11]. The combination of GDM and hyperglycemia during pregnancy has been confirmed in more than 20 million women globally [12]. Diagnosed GDM is associated with a seven-fold increased risk of developing T2DM and a two-fold increased risk of developing CVDs in the future. Additionally, women with GDM can develop cardiometabolic risk factors, such as obesity, dyslipidemia, MetS, and hypertension (HTN) [13]. Anxiety, depression, and stress, particularly psychological stress is associated with GDM, and depression is a known adverse effect [14]. Wu et al. [15] described the relationship between non-alcoholic fatty liver syndrome (NAFLD) and GDM via insulin resistance and the development of dysglycemia mid-pregnancy. Both insulin and hypoglycemic medications are used in the clinical treatment of women with GDM during pregnancy [16].

The prevalence of GDM in Saudi women is 19.6%; almost one-fifth of the pregnant women in Saudi Arabia have GDM because of dietary habits, overdriven metabolism during pregnancy, exacerbation of glucose tolerance, and a family history of diabetes that leads to GDM. However, the prevalence of GDM was 13.5% in Bahrain, 9.7–12.9% in Bangladesh, 8.1–9.3% in China, 10.5–15.7% in India, and 9.3–41.9% in Iran [17]. Saudi women with GDM and maternal DM have a 5.7 times and 2.8 times higher risk of maternal obesity, respectively. Additionally, intrauterine exposure to GDM/maternal DM is strongly associated with T2DM. Obesity, a sedentary lifestyle, and poor nutritional habits are considered non-modifiable risk factors in Saudi Arabia, as well as in the global population [18].

Vitamin D deficiency occurs due to low exposure to sunlight and aging. A lack of vitamin D can compromise bone metabolism and calcium absorption, leading to skeletal and non-skeletal diseases, such as diabetes and CVD [19]. In humans, vitamin D is formed in the skin, and cholecalciferol (vitamin D3) is generated by the photochemical conversion of 7-dehydrocholesterol. Vitamin D3 is converted by hepatic and renal enzymes into 25-hydroxyvitamin D (25(OH)D), the major storage and circulating form of vitamin D, and then into 1, 25-dihydroxyvitamin D, the hormonal form of vitamin D [20]. The deficiency values for 25-hydroxy vitamin D (25(OH)D) are those <30 nmol/L, insufficient values are those between 30–50 nmol/L, and sufficient levels are those <50 nmol/L [21]. Limited studies have been conducted on Saudi women with vitamin D deficiency, with and without pregnancy [22,23,24,25,26,27].

Single nucleotide polymorphisms (SNPs) are the most predominant type of genetic variation and are related to nucleotide amendments at any genomic position. Over 10 million SNPs have been associated with human diseases, including obesity, T2DM, and CVD [28]. In humans, SNPs can be used to predict diabetic diseases, including GDM [15]. Previous studies have confirmed that all patients, including women with GDM and T2DM, share a similar pathophysiology with impaired insulin secretion and increased insulin resistance, and that both diseases share common SNPs with similar degrees of size effects in similar risk alleles [29]. Previous studies have linked GDM and limited SNPs [30,31,32]. A limited number of SNPs are associated with GDM, including a similar association with T2DM; however, the ideal biomarker for predicting or detecting diabetes during pregnancy has not been confirmed. This is because the biological mechanisms underlying GDM in women are poorly understood. In this context, vitamin D deficiency and its molecular role in insulin resistance have been evaluated. The global prevalence of vitamin D deficiency is 46–87%, indicating that vitamin D deficiency might be a common health problem during pregnancy, including GDM [33]. Overall, vitamin D deficiency in Saudi Arabia increased from 30% to 100% between 1983 and 2015 in the general population and, to validate this, Al-Alyani et al. [34] performed a meta-analysis using previously published studies (2008–2015) in Saudi Arabia and revealed that the prevalence of vitamin D deficiency was approximately 60% [34].

Bone mineralization and metabolism are not possible without vitamin D, an essential steroid prohormone. It regulates both serum calcium and phosphate levels and influences skeletal development, skeletal architecture maintenance, hormone production, and immunological function [35]. Vitamin D triggers its biological effects by interacting with its receptor, i.e., the vitamin D receptor (VDR). Hereditary vitamin D-resistant rickets are caused by several loss-of-function SNPs in the *VDR* gene. In addition, the *VDR* gene has numerous modest allelic polymorphisms that have been associated with human diseases. The *VDR* gene is present on the 12th chromosome, in the quinine region 12–14 and located within 11 exons in the 12q12-14 region. The *VDR* encodes 427 amino acid proteins and spans 75 Kb [36]. Among the *VDR* genes, ApaI (rs79785232), BsmI (rs1544410), FokI (rs2228570), and TaqI (rs731236) SNPs have been commonly documented in human diseases. The *VDR* gene’s 3′ untranslated region (UTR) is where the ApaI SNP was found. The *VDR* has an intron containing the BsmI SNP. The FokI SNP in the *VDR* produces two protein isoforms with different durations through alternative splicing of the VDR mRNA. A longer protein is associated with the “F” allele, whereas a shorter protein is associated with the “f” allele. The longer isoform (FF genotype) may be more transcriptionally active, which may modify vitamin D signaling and response. The TaqI SNP is found in the intronic position of the *VDR* gene.

Bone mineral density (BMD), bone turnover, and susceptibility to osteoporosis have been previously studied. Both cancer risk and immune-related diseases have been investigated in relation to TaqI polymorphisms. Bone mineral density, osteoporosis, cancer, autoimmune diseases, and CVD are the only diseases associated with these SNPs [37,38,39,40]. The *VDR* is significantly expressed in pancreatic β-cells, and studies have linked SNPs in the *VDR* gene to insulin resistance and insulin secretory capacity [41,42]. The VDR is involved in a wide range of physiological processes, including glucose metabolism and insulin sensitivity. In Saudi Arabia, few studies have evaluated BsmI and Fok1 SNPs in the *VDR* [43,44,45]. This study investigated ApaI: rs79785232, BsmI: rs1544410, FokI: rs2228570, and TaqI: rs731236 SNPs in the *VDR,* as well as vitamin D serum levels in Saudi women diagnosed with GDM.

## 2. Materials and Methods

### 2.1. Ethical Concerns

This study was approved by the Institutional Review Board in the College of Medicine at King Saud University (KSU), under project number E-22-7318. A total of 180 Saudi women signed an informed consent form. This study was conducted in accordance with the Declaration of Helsinki.

### 2.2. Patients

In this case-control hospital-based study, 180 Saudi women were enrolled, including 90 women with GDM and 90 without (controls). All the women were enrolled at an outpatient clinic located on the second floor of the new building at King Khalid University Hospital. All pregnant women at 24–28 weeks visited the outpatient clinic during a routine checkup, with overnight fasting for a minimum of 8–10 h. Along with routine biochemical tests and a follow-up with clinicians, a 75 g oral glucose tolerance test (OGTT) was recommended for all pregnant women. During the same period, healthy women with normal OGTT levels were also recruited. The nominal threshold values were ≥5.1 mmol/L for fasting plasma glucose (FPG), ≥10.0 mmol/L for OGTT-1hPG, and ≥8.5 mmol/L for OGTT-2hPG [46]. The GDM diagnoses were confirmed based on studies by Zeng et al. [47] and Liang et al. [48]. Women with normal glucose levels in the OGTT were considered healthy, and women whose OGTT levels were high among the FPG in the first or second hour were diagnosed with GDM [49]. The inclusion criteria for women with GDM were based on elevated FPG and OGTT-2h, and these women were excluded from the control group. The exclusion criterion for women with GDM was normal glucose levels, and a similar criterion was used for the inclusion of women without GDM. In this study, non-Saudi women were excluded, along with Saudi women who did not sign the patient consent form or provide blood samples prior to enrollment.

### 2.3. Sample Size Calculation

We enrolled 90 GDM and 90 non-GDM pregnant women with and without GDM, based on the recommendations by Pourhoseingholi et al. [50].

### 2.4. Data Collection

Different data were collected from GDM and healthy women. Age was recorded, along with weight (kg) and height (cm), which were used to calculate the body mass index (BMI), based on the World Health Organization (WHO) criteria (kg/m^2^) [51]. During the second or third trimester, the weight, height, BMI, and blood pressure of the pregnant women who visited the outpatient clinic for routine checkups were monitored. Based on the AAMI/ESH/ISO protocol guidelines, HTN was evaluated in pregnant women using systolic (SBP) and diastolic blood pressure (DBP). The reference values for SBP and DBP were 140 mmHg and 90 mmHg, respectively [52].

### 2.5. Blood and Serum Analysis

We retrieved 2 mL of serum and 2 mL of blood in ethylenediaminetetraacetic acid (EDTA) tubes from each woman. Serum samples were used to analyze the lipid profile parameters, EDTA blood was used to estimate hemoglobin (Hb)A1c for a minimum of 3 months, and additional EDTA blood was used for molecular analyses. Using Roche Diagnostics kits (Roche Diagnostics, Rotkreuz, Switzerland) with Cobas e411 automated sequencing equipment, the triglycerides (TG), total cholesterol (TC), and high-density lipoprotein cholesterol (HDLc) levels were measured. The low-density lipoprotein cholesterol (LDLc) levels were manually measured [53]. The HbA1c levels were measured using a Roche kit, based on the American Diabetes Association criteria. Therefore, HbA1c < 5.7% was considered normal, 5.7–6.4% was considered prediabetic, and >6.5% was considered diabetic [54]. Finally, the total serum 25-hydroxyvitamin D or serum 25-(OH)-D_3_ and vitamin D levels were measured in women with GDM using a Roche Diagnostics kit and an automated analyzer. Vitamin D deficiency was confirmed with values <30 nmol/L, 30–50 nmol/L was considered insufficient, and >50 nmol/L was considered sufficient [20,27]. In this study, the FPG, postprandial blood glucose (PPBG), OGTT-1h, and OGTT-2h values were obtained from medical records on the hospital’s premises after confirming the GDM women and controls.

### 2.6. Molecular Analysis for the VDR Gene

**Figure 1:**
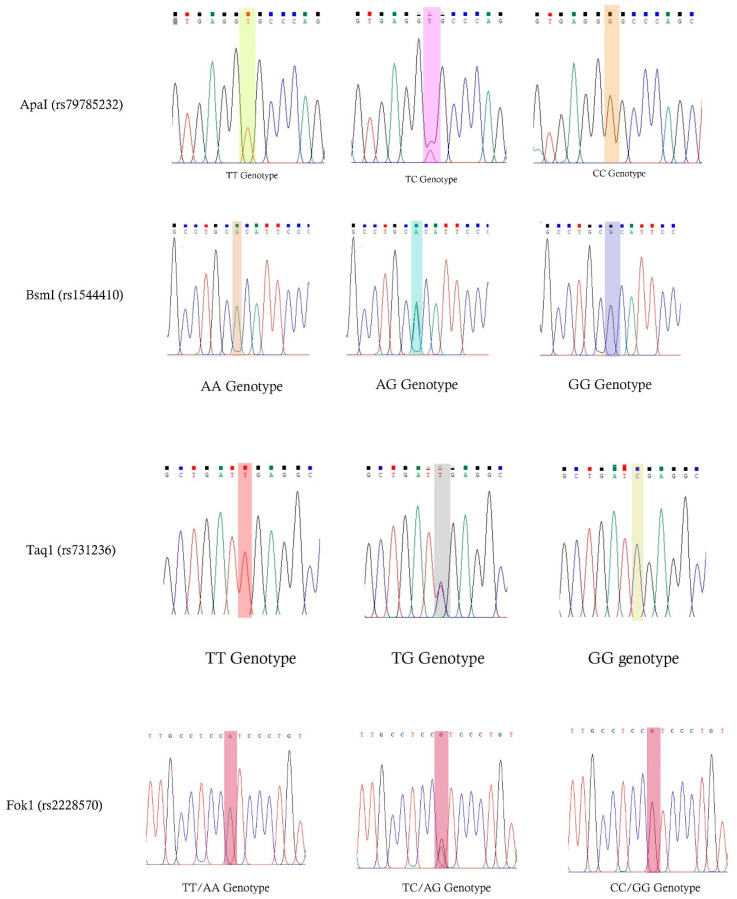
Sanger sequencing analysis represents the chromatograms of the ApaI, BsmI, FokI, and TaqI SNPs in the *VDR* gene. Different color indicates the exchange of nucleotide towards a specific SNPs.

Using 150 µL of peripheral EDTA blood, genomic DNA was extracted using the Qiagen DNA isolation kit, according to the manufacturer’s instructions. Red (RBC) and white blood cell (WBC) lysis, along with protein purification and DNA extraction, was completed in 35 min using centrifugation at 3000 rpm and 15,000 rpm, and incubation at 56 °C for 10 min. Finally, 200 µL of genomic DNA was obtained from 150 µL of blood. The DNA was stored at −80 °C until analysis. DNA quantification was performed using a NanoDrop spectrophotometer (NanoDrop Spectrophotometer, Thermo Scientific, Waltham, MA, USA) and the concentration of DNA was converted into 20 µg/mL and used for the amplification screening program for ApaI, BsmI, FokI, and TaqI SNPs in the *VDR* gene. The polymerase chain reaction (PCR) was performed using the Qiagen master mix (Hilden, Germany), i.e., 10× Buffer, MgCl_2_, Taq DNA polymerase, 10 pmol of primers for each SNP, DNA, and double-distilled water to finalize the reaction volume at 50 µL. Normal Applied Biosystems’ (Waltham, MA, USA) thermal cyclers were used to run the PCR, with the initial denaturation at 95 °C (5 min); 35 cycles of denaturation at 95 °C (30 s), annealing at 66–68 °C (30 s), an extension at 72 °C (45 s); and a final extension at 72 °C (5 min); and a hold at 4 °C. Further details are described in Table 1. All 180 samples were assigned a code. The PCR durations were 1.20–1.35 h. The unpurified and undigested PCR products were run on a 2% agarose gel, including a 100 bp DNA marker from Thermo Fisher Scientific (USA), stained with 10 U of ethidium bromide and visualized using ultraviolet imaging (UVI). Specific New England Biolabs (NEB, Ipswich, MA, USA) restriction enzymes were used for precise SNPs (Table 1), and reactions were incubated for 18 h at 37 °C. The PCR/RFLP data were recorded using a gel documentation system, and the PCR products for the four SNPs were purified and subjected to Sanger sequencing analysis, as described in our previous study. Complete molecular analysis was performed in the G-141 laboratory and Sanger sequencing was performed outside the G-141 laboratory. From the outset, we received Fasta, ABI, and PDF files, and the genotyping data were analyzed based on the band size and specific markers (Figure 1).

### 2.7. Statistical Analysis

Different statistical software packages were used to calculate the different statistical models. Initially, both clinical and genotype data were recorded in an Excel 2019 spreadsheet after labelling the codes. Categorical variables were reported as frequencies and percentages, while numerical variables were reported as mean ± standard deviation. Using the Mann–Whitney U test, continuous data were analyzed, while categorical data were calculated using the chi-square (ꭓ^2^) or Fisher’s exact test. The SNPStats software [55] was used to calculate the Hardy–Weinberg equilibrium (HWE), odds ratios (ORs), 95% confidence intervals (95% CIs), and *p*-values. In addition, a haplotype analysis was performed. HWE analysis was performed for the GDM and control groups using chi-square (ꭓ^2^) tests. Serum levels of vitamin D were measured in pregnant women using Fisher’s exact test, and the Mann–Whitney U test was performed while measuring the vitamin D levels and SNPs. Multiple linear logistic regression models were used in SPSS to evaluate the GDM covariates and the ApaI (rs79785232), BsmI (rs1544410), FokI (rs2228570), and TaqI (rs731236) SNPs in the *VDR* gene. One-way analysis of variance (ANOVA) was carried out in the Jamovi software [56] to compare the GDM covariates, the types of vitamin D levels, the SNP genotypes, and the combination of GDM covariates and the four SNP genotypes. Crude ORs and 95% CIs for both groups of SNPs present in the *VDR* were calculated using a logistic regression model. Haploview (version 4.2) was used to examine the linkage disequilibrium (LD) and coefficient (D’) in the pregnant women. A generalized multifactor dimensionality reduction (GMDR) model with four SNPs was used to investigate the gene–gene interactions, dendrograms, and graphical depletion models between the pregnant women and selected demographic features. Statistical significance was considered when *p* < 0.05.

## 3. Results

### 3.1. Characteristics of the Studied Traits in Saudi Women

The characteristic features of the GDM and control groups are shown in Table 2. The mean age was 30.30 ± 6.25 (18–45) years. The mean ages in the GDM and control groups were 32.34 ± 5.42 (21–44) and 28.26 ± 6.39 (18–39) years, respectively. The pre-pregnancy weight (76.75 ± 12.85 vs. 73.57 ± 12.38; *p* = 0.092), height (158.03 ± 5.84 vs. 157.86 ± 5.11; *p* = 0.835), and BMI (30.60 ± 4.87 vs. 29.39 ± 4.38; *p* = 0.081) were high among the women with GDM compared to the averages of those without GDM, although there was no significant association (*p* > 0.05). Women with GDM exhibited higher levels and showed positive associations for SBP (130.20 ± 11.95 vs. 120.77 ± 6.10; *p* < 0.0001), DBP (81.47 ± 7.84 vs. 78.00 ± 5.02; *p* = 0.0005), FPG (5.54 ± 1.69 vs. 4.55 ± 0.62; *p* = 0.0004), PPBG (8.51 ± 17.63 vs. 4.79 ± 0.83; *p* < 0.0001), OGTT-1h (11.45 ± 1.42 vs. 7.05 ± 1.46; *p* < 0.0001), OGTT-2 h (9.81 ± 1.58 vs 6.31 ± 1.54; *p* < 0.0001), and HbA1c levels (5.59 ± 0.52 vs. 5.26 ± 0.33; *p* = 0.0008), when compared with the levels of those without GDM. Additionally, the serum parameters, such as the TG (1.87 ± 1.07 vs. 1.54 ± 2.11; *p* = 0.187), TC (5.39 ± 1.14 vs. 5.70 ± 1.27; *p* = 0.086), and LDLc levels (3.83 ± 0.86 vs. 3.71 ± 0.98; *p* = 0.383) were different between the GDM and control groups (*p* > 0.05). Furthermore, the serum levels of vitamin D were similar (*p* = 0.391) between the GDM (47.83 ± 21.23) and control groups (50.51 ± 20.59). Finally, a family history of GDM (*p* = 0.087) or HTN (*p* = 0.966) was not significant in this study. Healthy women had elevated levels of HDLc compared to those with GDM (i.e., 0.92 ± 0.44 vs. 0.71 ± 0.36; *p* = 0.0005).

### 3.2. Interaction of the Studied SNPs with the VDR Gene in Pregnant Women

The allele (Table 3), genotype, and genetic models (Table 4) of the defined frequencies of the ApaI/rs79785232, BsmI/rs1544410, FokI/rs2228570, and TaqI/rs731236 SNPs in the *VDR* gene are presented in the tables, including the statistical analysis. Table 3 defines the statistical analysis between the GDM and control groups for all the studied SNPs in the *VDR* gene. The A allele (72.8% vs. 64.4%) in the ApaI SNP was high in healthy women, whereas the C allele (35.6% vs. 27.2%) was high in women with GDM. Overall, the statistical analysis showed a non-significant association for the ApaI allele (C vs. A: OR-2.47 (95%CI: 0.94–42.31); *p* = 0.088). A similar association was found in the C and T alleles in the FokI SNP when comparing the GDM (79.4% and 20.6%) and control groups (81.1% and 18.9%) (T vs. C: OR-1.11 (95%CI: 0.66–1.86); *p* = 0.691). However, the BsmI and TaqI SNPs showed positive frequencies among the allele frequencies (G vs. A: OR-2.10 (95%CI: 1.35–3.24); *p* = 0.0009 and C vs. T: OR-1.83 (95%CI: 1.13–2.96); *p* = 0.012). The allele frequencies of the A and G alleles in the GDM and control groups were 71.1%/28.9% and 73.3%/26.7%, respectively, in the BsmI SNP. The frequencies of T and C were 67.8%/32.2% and 79.4%/20.6% in the GDM and control groups.

Table 4 describes the HWE analysis, genotype, and genetic models of the ApaI to TaqI SNPs present in the *VDR*. The HWE analysis evaluated in the *VDR* of healthy women was inconsistent with the ApaI (ꭓ^2^ = 0.03; *p* = 0.85), BsmI (ꭓ^2^ = 1.67; *p* = 0.19), FokI (ꭓ^2^ = 1.51; *p* = 0.21), and TaqI (ꭓ^2^ = 0.01; *p* = 0.89) SNPs. However, a similar pattern was found in the GDM group with the ApaI (ꭓ^2^ = 1.19; *p* = 0.27), BsmI (ꭓ^2^ = 0.81; *p* = 0.36), FokI (ꭓ^2^ = 2.01; *p* = 0.15), and TaqI (ꭓ^2^ = 0.63; *p* = 0.42) SNPs.

The genotype frequencies of AA, AC, and CC of the ApaI SNPs were 38.9%, 51.1%, and 10% in the GDM group, and 54.4%, 36.7%, and 8.9% in the control group, respectively. None of the genotypes (AC vs. AA: OR-1.95 (95%CI: 1.04–3.63); *p* = 0.034; CC vs. AA: OR-1.47 (95%CI: 0.51–4.22); *p* = 0.463) or the different genetic models (AC + CC vs. AA: OR-1.87 (95%CI: 1.03–3.39); *p* = 0.036; AA + CC vs. AC: OR-0.55 (95%CI: 0.30–1.01); *p* = 0.050; and CC vs. AC + AA-OR, 1.13 (95%CI: 0.41–3.09); *p* = 0.798) showed a positive association. For the BsmI SNP, 34.4%, 44.5%, and 21.1% of the AA, AG, and GG genotypes were present in women with GDM, and 51.1%, 44.5%, and 4.4% were present in women without GDM, respectively. The GG genotype (AG vs. GG: OR-7.04 (95%CI: 2.18–22.72); *p* = 0.0003), dominant (AG + GG vs. AA: OR-1.99 (95%CI: 1.09–3.62); *p* = 0.023), and recessive models (AG + GG vs. AA: OR-5.75 (95%CI: 1.87–17.69); *p* = 0.0008) showed a positive association in comparisons between the GDM and control groups. The genetic models did not show any significant association for the FokI SNP in comparisons between the GDM and control groups (CT vs. CC: OR-1.07 (95%CI: 0.55–2.09); *p* = 0.826; TT vs. CC: OR-1.24 (95%CI: 0.35–4.28); *p* = 0.099; CT + TT vs. CC: OR-1.10 (95%CI: 0.59–2.05); *p* = 0.751; CC + TT vs. CT: OR-0.94 (95%CI: 0.49–1.82); *p* = 0.867; CC vs. CT + TT: OR-1.92 (95%CI: 0.85–4.31); *p* = 0.109). The obtained CC, CT, and TT genotypes were 65.5%, 27.8%, and 6.7% in the GDM, and 67.8%, 26.7%, and 5.5% in the controls, respectively. The final SNP in this study was TaqI, which had 47.8%, 40%, and 12.2% in the GDM and 63.3%, 32.2%, and 4.5% in the controls for the TT, TC, and CC genotypes, respectively. The CC genotype (CC vs. TT: OR-3.64 (95%CI: 1.08–12.23); *p* = 0.028) and dominant model (TC + CC vs. TT: OR-1.88 (95%CI: 1.04–3.42); *p* = 0.035) showed a strong association for the TaqI SNP. Additionally, the TC genotype (TC vs. TT: OR-1.64 (95%CI: 0.87–3.08); *p* = 0.119), co-dominant (CC + TT vs. TC: OR-0.71 (95%CI: 0.38–1.31); *p* = 0.278), and recessive models (CC vs. TC + TT: OR-2.99 (95%CI: 0.91–9.78); *p* = 0.059) showed a negative association for this SNP.

### 3.3. Serum Levels of Vitamin D along with the BMI Levels in Pregnant Women Groups

In this case-control study, serum levels of vitamin D were evaluated in women with and without GDM. The overall mean levels of vitamin D were 47.83 ± 21.23 nmol/L in the GDM and 50.51 ± 20.59 nmol/L in the controls. Vitamin D levels were divided into deficient (<30 nmol/L), insufficient (30–50 nmol/L), and sufficient (>50 nmol/L) levels, as shown in Table 5. The BMI levels were categorized into four groups, i.e., normal, overweight, obese, and morbidly obese. In the GDM group, normal, overweight, obese, and morbidly obese values were found to be 14.4%, 27.8%, 41.1%, and 16.7%, respectively, and 16.7%, 34.4%, 42.2%, and 6.7% for the non-GDM group. The chi-square test was implemented to determine the relationship between GDM status and vitamin D and the BMI levels in pregnant women. The chi-square test demonstrated that the vitamin D levels (ꭓ^2^ = 2.52, *p* = 0.28) and BMI levels (ꭓ^2^ = 4.65, *p* = 0.19) in pregnant women were independent of the GDM status (i.e., GDM and non-GDM). This suggests that deviations in the vitamin D and BMI levels are unrelated to the GDM status.

### 3.4. Comparison of Vitamin D Levels with Baseline Characteristics

Table 6 shows the comparison between 14 covariates and the characterization of vitamin D levels for the three groups, as described in Table 5. Age, weight, BMI, SBP, DBP, the levels of FPG, PPBG, OGTT-1h, OGTT-2h, HbA1c, TG, TC, HDLc, and LDLc were the considered covariates in this study. A one-way ANOVA was performed between these 14 covariates and the three groups of vitamin D levels: deficient, insufficient, and sufficient. The deficient values of the elevated levels were found in regard to the age (33.42 ± 5.06), DBP (82.79 ± 7.92), TC (5.70 ± 1.23), HDLc (0.77 ± 0.43), and LDLc levels (4.09 ± 0.97). However, the HbA1c (5.65 ± 0.49) and TG (2.13 ± 1.15) levels were high in the insufficient category. Finally, the weight (77.39 ± 12.07), BMI (31.05 ± 4.92), SBP (130.82 ± 12.38), FPG (5.87 ± 1.91), PPBG (10.81 ± 26.99), OGTT-1h (11.58 ±1.73), and OGTT-2h (10.01 ± 1.88) were found to be high in the sufficient category. The ANOVA confirmed that the FPG (*p* = 0.0001), PPBG (*p* < 0.0001), OGTT-1h (*p* = 0.005), HDLc (*p* = 0.022), and LDLc (*p* = 0.001) levels were positively correlated. This study also confirmed that there were no significant differences in regard to the age, weight, BMI, SBP, DBP, OGTT-2h, HbA1c, TG, and TC (*p* < 0.05), according to the different vitamin D levels in the GDM group.

### 3.5. Relationship between the Vitamin D Levels and VDR SNPs in Women with GDM

In this study, we compared the vitamin D serum levels, which were shown to be deficient, insufficient, and sufficient, with the normal homozygous (XX), heterozygous (XY), and homozygous variants (YY) of ApaI, BsmI, FokI, and TaqI SNPs in the *VDR* gene (Table 7). The AA, AC, and CC genotypes were measured within the deficient, insufficient, and sufficient categories for the ApaI SNP and were associated with sufficient levels (*p* < 0.0001). A similar pattern for the three genotypes was measured among the BsmI (*p* = 0.016), FokI (*p* = 0.001), and TaqI (*p* = 0.045) SNPs, and insufficient levels were associated with these genotypes among women with GDM.

### 3.6. Regression Model of the GDM Covariates and SNPs in the VDR Gene

In this study, a multiple logistic regression model was used to study 15 covariates and the ApaI–TaqI SNPs in the *VDR*, as shown in Table 8. The 15 covariates included age, weight, BMI, SBP, DBP, as well as the FPG, PPBG, OGTT-1h, OGTT-2h, HbA1c, TG, TC, HDLc, LDLc, and vitamin D levels. Multiple logistic regression analysis confirmed that OGTT-1h (*p* = 0.005) was associated with four SNPs in the *VDR* gene.

### 3.7. ANOVA of the GDM Covariates and SNPs in the VDR Gene

A one-way ANOVA was performed for the GDM covariates and SNPs present in the *VDR* gene. The details are listed in Table 9. The elevated levels in ApaI SNP were the age (35.56 ± 3.88), DBP (84.22 ± 8.73), FPG (6.30 ± 3.22), PPBG (11.27 ± 28.10), TG (2.44 ± 1.14), TC (5.99 ± 0.93), and vitamin D (52.49 ± 19.32) levels. HbA1c (5.66 ± 0.57) was the only elevated level in the BsmI SNP. For the FokI SNP, the OGTT-1h (12.72 ± 2.41), OGTT-2h (10.40 ± 1.99), and HDLc (0.88 ± 0.07) levels were high. Finally, in the TaqI SNP, the weight (81.16 ± 16.56), BMI (31.75 ± 6.90), and SBP (131.09 ± 10.55) levels were high. The overall statistical analysis using ANOVA confirmed that the FPG and PPBG were strongly associated (*p* < 0.05) with ApaI, BsmI, FokI, and TaqI SNPs in the *VDR*. Additionally, the OGTT-2h (*p* = 0.048) and LDLc (*p* = 0.049) in the ApaI SNP and OGTT-1h (*p* = 0.045), TG (*p* = 0.017), TC (*p* = 0.034), and HDLc (*p* = 0.0005) were individually associated.

### 3.8. Haplotype Analysis of VDR SNPs

The results of the haplotype analyses of the ApaI, BsmI, FokI, and TaqI SNPs are shown in Table 10. The combination of these four SNPs resulted in 16 distinct genotypes with four nucleotides. The combination of AGCC (OR-5.30 (95%CI: 1.34–21.01); *p* = 0.019), CATT (OR-6.91 (95%CI: 0.78–32.19); *p* < 0.0001), CGTT (OR-12.1 (95%CI: 3.67–46.9); *p* < 0.0001), AGTC (OR-2.86 (95%CI: 0.81–16.75); *p* < 0.0001), and CATT (OR-4.30 (95%CI: 0.88–29.83); *p* < 0.0001) alleles showed a strong association in women with GDM. A higher risk (12.1-fold) was found for the CGTC haplotype.

### 3.9. Analysis of Linkage Disequilibrium (LD)

**Figure 2:**
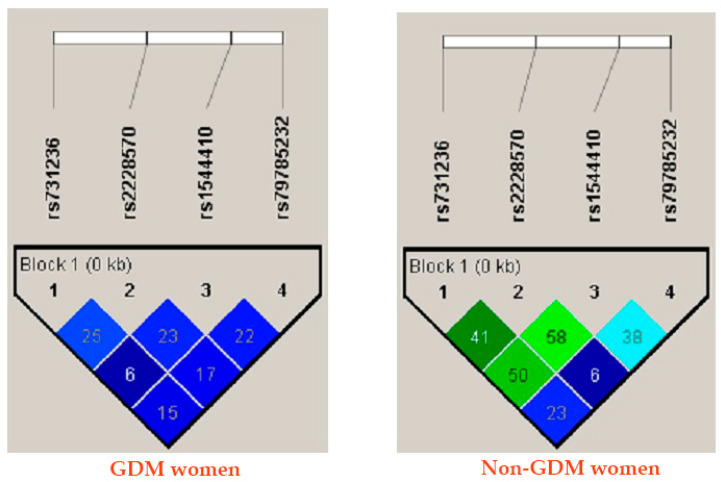
Linkage disequilibrium analysis between GDM/non-GDM women for the four SNPs studied in the *VDR* gene.

The LD was analyzed in both groups for ApaI, BsmI, FokI, and TaqI SNPs in the *VDR*, as shown in Table 11 and Figure 2. All the pregnant women had strong associations with the ApaI, BsmI, FokI, and TaqI SNPs in the LD analysis (Table 11 and Figure 2).

### 3.10. Analysis of the GMDR Model in GDM Women

**Figure 3:**
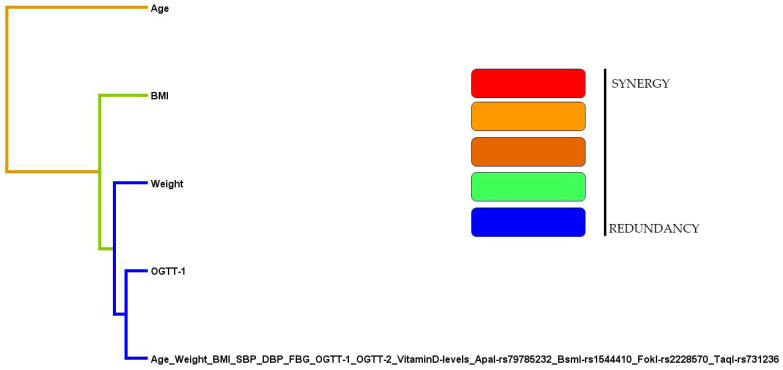
Analysis of dendrogram between GDM women and SNPs present in the *VDR* gene.

**Figure 4:**
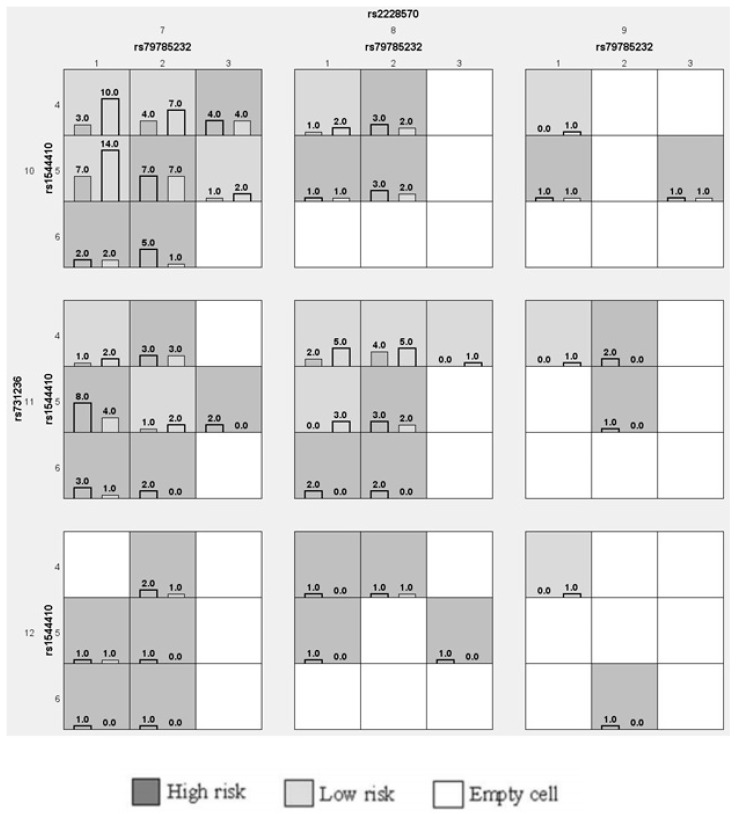
Graphical depletion model analyzing the predicted risk of four SNPs in GDM women.

A combination of genotypes, including ApaI, BsmI, FokI, and TaqI SNPs, among the pregnant women was created to study the gene–gene interactions (Table 12), dendrograms (Figure 3), and the graphical depletion/depiction method (Figure 4). Among the gene–gene interactions, the combination of rs1544410 (OR-2.10 (95%CI: 1.15–3.83); *p* = 0.015), rs1544410/rs731236 (OR-3.38 (95%CI: 1.75–6.51); *p* = 0.002), rs1544410/rs2228570/rs731236 (OR-3.96 (95%CI: 2.10–7.46); *p* < 0.001), and rs79785232/rs1544410/rs2228570/rs731236 (OR-4.81 (95%CI: 2.54–9.09); *p* < 0.001) showed a positive association. The dendrogram analysis is shown in Figure 3. We dissected the clinical and genotype data to study in detail the relationship between the *VDR* and pregnant women. We confirmed that age was strongly associated with the observed variables, as well as the BMI, weight, OGTT, and a combination of all factors, such as age, weight, BMI, SBP, DBP, as well as the FPG, PPBG, OGTT-1h, OGTT-2h, and vitamin D levels and the ApaI, BsmI, FokI, and TaqI SNP. However, no synergistic association was observed in this study. A graphical representation of the overall findings categorized the impact of the loci model as high and low risk. Furthermore, statistical interactions were defined by the GMDR model. Darker-shaded cells have higher-risk combinations, whereas lighter-shaded cells have lower-risk combinations. White/blank cells are genotype pairs for which no data are available. The bars depict the hypothetical case (left) and control (right) distributions for each multifactor combination. In this study, as shown in Figure 4, high-risk groups were identified for all possible combinations of the four SNPs. Furthermore, a combination of low- and no-risk groups was included. Using the GMDR model analysis, the combination of the gene–gene interaction, dendrogram, and graphical depiction methods revealed a positive association with the risk of the *VDR* genotypes in women with GDM.

## 4. Discussion

This study explored the association of four SNPs present in the *VDR* with susceptibility toward GDM and vitamin D serum levels in Saudi women. Prior to the start of this study, we confirmed that the selected sample size was adequate [50] and evaluated the principle of HWE analysis, which was fulfilled by the controls. All the SNP data in our study suggested that the alleles of all the variants tested were randomly separated from one generation to the next, and the sample was normal for the population studied [57]. GDM is a temporary form of diabetes that develops for the first time during pregnancy, as it is related to carbohydrate intolerance, and resolves after delivery of the baby [58]. Previous studies have also reported that vitamin D deficiency is a risk factor for GDM and that vitamin D [59] directly influences pancreatic β-cells and is required for optimal insulin secretion [60]. Genetic factors in GDM are associated with the regulation of insulin secretion and peripheral insulin sensitivity [61]. Vitamin D has essential immunoregulatory properties, and vitamin D deficiency may play a role in the development of T2DM by affecting insulin secretion [62]. Previous studies have shown a link between T2DM and the *VDR* [63] and have confirmed that vitamin D deficiency can increase the risk of mortality in women with GDM [64,65]. The *VDR* gene is implicated in the insulin metabolic pathway, and its polymorphism is linked to insulin resistance and secretion [66]. Additionally, the *VDR* gene is very important in Saudi Arabia, as almost 60% of the population exhibits a vitamin D deficiency [34]. There are few documented studies available in Saudi Arabia on GDM [43,44,45], and this is an update to the previous studies [43,44,45]. In this study, we opted for four risk factor SNPs (i.e., ApaI-rs79785232, BsmI-rs1544410, FokI-rs2228570, and TaqI-rs731236), along with vitamin D serum levels, in both patients with and without GDM.

In this case-control study, we investigated the molecular effects of four SNPs present in the *VDR* gene among women in Saudi Arabia. In addition to T2DM, obesity, and CVD, GDM progression to DM is described as a multifactorial and metabolic disorder characterized by carbohydrate intolerance, and SNPs in the *VDR* gene can influence the susceptibility of pregnant women to GDM. In addition, proteins present in the VDR may mediate the effects of vitamin D in the body. The current study results confirmed that age, HTN levels, glucose levels, and a family history of T2DM were strongly associated with GDM in women (*p* < 0.05). The HWE analysis was consistent with the results from this study (*p* > 0.05). The BsmI and TaqI SNPs were associated with alleles (*p* < 0.05), genotypes (*p* < 0.05), and different genetic models (*p* < 0.05), whereas the ApaI SNP was associated with the AC genotype and dominant model (*p* < 0.05). Vitamin D levels were associated with deficient levels (*p* = 0.0002), as well as with normal and overweight BMI levels (*p* = 0.0004). When vitamin D levels were measured with GDM covariates, the FPG (*p* = 0.0001), PPBG (*p* < 0.0001), OGTT-1h (*p* = 0.005), HDLc (*p* = 0.022), and LDLc (*p* = 0.001) levels were significantly different. However, there were no significant differences in the GDM group in terms of age, weight, BMI, SBP, DBP, OGTT-2h, HbA1c, TG, and TC (*p* > 0.05) based on different vitamin D levels. When similar vitamin D levels were measured in different genotypes, we confirmed that the ApaI SNP was associated with sufficient levels (*p* < 0.0001), whereas the BsmI (*p* = 0.016), FokI (*p* = 0.001), and TaqI (*p* = 0.045) SNP were associated with insufficient levels. The logistic regression model confirmed that OGTT-1h (*p* = 0.005) was strongly associated with GDM, whereas the ANOVA confirmed that FPG and PPBG (*p* < 0.05) were strongly associated with all four SNPs. Additionally, OGTT-2h (*p* = 0.048) and LDLc (*p* = 0.049) were associated with the ApaI, and the FokI SNP, OGTT-1h (*p* = 0.045), and lipid profile parameters (*p* < 0.05) were also associated. The haplotype analysis revealed positive associations among the examined SNPs, which seemed compatible with the hypothesis that variants and combinations of multiple SNP genotypes enhance the risk of GDM in women. The haplotype analysis revealed that different combinations of alleles, including AGCC (*p* = 0.019), CATT (*p* < 0.0001), CGTC (*p* < 0.0001), AGTC (*p* < 0.0001), and CATT (*p* < 0.0001), were strongly associated. The LD analysis was strongly associated (*p* < 0.05) with all the combinations, as shown in Table 11. Among the gene–gene interactions, the combinations of rs79785232/rs1544410/rs2228570/rs731236 (*p* < 0.001), rs1544410 (*p* = 0.015), rs1544410/rs2228570/rs731236 (*p* < 0.001), and rs1544410/rs731236 (*p* = 0.002) were positively associated. Four potential LD blocks were constructed using the four SNPs in the *VDR* gene (Figure 2). The dendrogram analysis revealed an intermediate association between GDM and the VDR SNPs. Finally, the depletion model showed all possible combinations, and the overall GMDR analysis confirmed a positive association with the genotypes present in the *VDR* SNPs. Overall, the statistical analysis confirmed that SNPs in the VDR play a role in the development of GDM in Saudi women.

Various SNPs in the *VDR* have been studied in GDM, and both positive [43,61,67,68,69,70,71,72,73,74,75] and negative associations [76,77,78,79,80] have been reported. In our study, the ApaI, BsmI, and FokI SNPs were associated, and our findings were consistent with previous studies [43,61,67,68,69,70,71,72,73,74,75]. The TaqI SNP was not associated, which is consistent with previous studies [68,71,72,78,79]; however, some of the studies were inconsistent [43,61,67,69,70,73,74] with our study. There have been meta-analyses involving distinct SNPs in the *VDR* gene with GDM [33,62,81,82]. Meta-analysis studies by Liu et al. [81] confirmed that both the ApaI and FokI SNPs are associated and recommended screening for them as biomarkers for GDM in women. Zhou et al. [33] confirmed that the ApaI SNP was associated with ApaI. Another meta-analysis by Zeng et al. [62] confirmed that the BsmI (rs739837) SNP was associated with GDM, and Wang et al. [82] confirmed that the FokI SNP may confer susceptibility to GDM. In addition, meta-analysis studies have documented other human diseases, such as T2DM [63], obesity [83], CVD [84], and polycystic ovary syndrome (PCOS) [85], which are all risk factors for GDM and showed associations with specific SNPs in the *VDR* gene [63,83,84,85].

Low levels of vitamin D constitute an important environmental risk factor for the development of human diseases [86]. Vitamin D deficiency was found in 74% of the pregnant women [87]. Vitamin D deficiency may lead to immune system malfunction and increased risk of cancer, CVD, diabetes, rheumatic sickness, muscle weakness, chronic pain, and neuropsychiatric dysfunction. A lack of vitamin D during pregnancy causes infantile rickets, which has also been associated with decreased prenatal growth and delayed neonatal development. Vitamin D deficiency in pregnant women may lead to GDM [88]. Vitamin D screening is common in Saudi women. In our study, women with GDM had lower vitamin D levels when compared with healthy women. The serum vitamin D levels in this study were consistent with those in similar studies carried out in women with GDM [89,90], although the findings of some studies were inconsistent [91,92,93,94]. Our findings were in agreement with those of meta-analyses studies [95,96,97]. A meta-analysis study by Milajerdi et al. [98] confirmed from 29 prospective and nested case-control studies that 26% of pregnant women with a vitamin D deficiency had increased risk of GDM. Amraei et al. [99] confirmed that vitamin D deficiency may increase the risk of GDM. A recent meta-analysis by Wu et al. [100] confirmed that vitamin D supplementation improves blood lipid levels in women with GDM.

The biological activity of vitamin D is controlled by its interaction with the vitamin D3 receptor protein. A vitamin D deficit or SNPs in the *VDR* gene may disrupt the vitamin D pathway [101]. Similar studies conducted in Saudi women have confirmed low serum vitamin D levels. Al-Ajlan et al. [22] confirmed the low serum levels in women with GDM. One previous study on pregnant Saudi women confirmed vitamin D deficiency among 50% of the participants and vitamin D inefficiency among 43.8% [23]. Another study, conducted in Riyadh, confirmed that 60% of women were deficient in vitamin D [20]. Almidani et al. [102] confirmed that 87.4% of mothers had a vitamin D deficiency during pregnancy. A study from the Al-Jouf region confirmed that 70% of pregnant women are deficient in vitamin D [103]. Women with PCOS in Saudi Arabia also have low vitamin D levels [27]. Al-Ayani et al. [34] confirmed that 60% of the Saudi population are deficient. Based on previous studies, the majority of studies in Saudi Arabia among Saudi women showing vitamin D deficiency suggest that the deficiency is due to their conservative clothing habits, preventing them from exposing much of their skin to sunlight [104].

The *VDR* gene contains 14 exons. The promoter region is present on exon-1 and has six variants (a–f), and exons 2–9 are present in the coding region. These four SNPs may influence the receptor structure and regulate the response to vitamin D. The ApaI-rs7975232 SNP was present in the intron-8 region, located at the 3′ UTR region and appeared in 12:47845054 (GRCh38) with alterations (A > C or A > a). It has no functional effects and may affect mRNA stability. The BsmI-rs1544410 SNP was also present in the 3′ UTR region, appeared on intron-8, and at 12:47879112 (GRCh38) with substitution in A > G/C > T or B > b. The FokI-rs2228570 SNP appears in the second exon at 12:47879112 (GRCh38), C > T or F > f, and plays a role in post-transcriptional processes. It is known to be a transition polymorphic site located in the start codon of the *VDR* gene, which affects both the amino acid amendments (Met-1-Thr) and the function of the encoded receptor protein, causing the production of long and short VDR protein variants. The short form has a longer transcriptional functional activation capacity than the long VDR form. The TaqI-rs731236 SNP was present on exon-9 and existed at 12:47844974 (GRCh38) with C > T or T > t. It affects mRNA stability, which influences the biological function of vitamin D. The ApaI, BsmI, and TaqI SNPs are present in the 3′ UTR region and frequently exhibit an LD effect [105]. The FokI SNP is an independent polymorphic site and does not exist in LD analysis along with the other SNPs present in the *VDR* gene [106]. These four SNPs may affect the mRNA stability and transcription rate, altering the expression, length, and activity of the protein correlated with disease risk and severity in humans [107]. *VDR* SNP studies were conducted in Saudi Arabia related to different human diseases, including in children, adults, men, and women. These studies showed all forms of associations [43,67,108,109,110,111,112,113,114,115,116,117,118,119,120,121,122,123,124,125,126,127,128,129,130,131,132,133,134,135,136,137,138,139,140,141,142,143]. The overall study results confirmed the varied analysis of specific diseases based on environmental factors, and variations in vitamin D can be considered a major issue.

Vitamin D deficiency is caused by inadequate exposure to sunlight and is a common health problem. In addition to sunlight, dairy products and fatty fish can be used as alternatives to mitigate vitamin D deficiency. Approximately 1 billion individuals suffer from vitamin D insufficiency globally. In Saudi Arabia, where it is always humid and has very high temperatures, especially in the summer, vitamin D deficiency should be very rare. However, because Saudi men wear traditional clothes that cover almost their entire body and spend most of their day indoors, they do not get enough sunlight. Therefore, a lack of vitamin D is a common health problem in Saudi adults, especially among women and younger adults. However, studies conducted in various parts of Saudi Arabia have shown that the incidence varies [144]. This is why serum and molecular studies on vitamin D, as well as the *VDR*, show variable results within the kingdom. Limited studies have been conducted in Saudi women diagnosed with GDM, and SNPs in the *VDR* gene [43,44,45] and serum levels [22,34,102,145] were studied separately. In the current study, we integrated serum levels and SNPs of the *VDR* gene and presented a combined treatment in women with GDM. Based on our data, we recommend screening for the *VDR* gene, along with vitamin D serum levels, using a large sample size in future studies to rule out deficiencies in individuals in Saudi Arabia.

**Figure 5:**
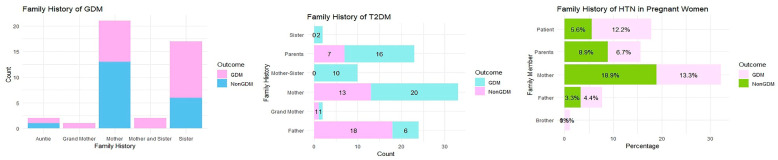
Presence of family history of GDM, T2DM, and HTN in both GDM and non-GDM women.

In this study, family histories of GDM, T2DM, and HTN were documented in patients with and without GDM, as shown in Figure 5. Overall, 47.8% of women with GDM had sisters with GDM, 34.8% had mothers who had been previously diagnosed with GDM, 8.7% had both a mother and older sisters that had GDM confirmed during their pregnancy, and 4.3% had aunts and grandmothers who had GDM during their pregnancy. Among women without GDM, 65% had mothers who had previously had GDM confirmed during their pregnancies, and 30% had sisters and 5% had aunts who had GDM during their initial pregnancies. The family histories of T2DM in women with GDM were 2.2% in a grandmother and mother with the older daughter (sister), 13% in fathers, 43.5% in mothers, and 34.8% in the parents. In healthy women, 2.2% of those with a family history of T2DM was found in the grandmother, 17.9% in the parents, 33.3% in the mother, and 46.2% in the father.

The family history of HTN was 37.8% in women with GDM and 36.7% in those without GDM. Regarding women with GDM, 2.9% of the family history was found in the brother, 11.8% in the father, 17.6% in the parents, 32.4% in the patient, and 35.3% in the mother. Among healthy women, 9.1% of the family history was found in the father, 15.2% in the patient, 24.2% in the parents, and 51.5% in the mother. On average, the highest prevalence of family history was found in mothers with GDM (*n* = 34.8%), T2DM (33.3%), HTN (35.3%), and in healthy women. Family histories were 65% in GDM, 43.5% in T2DM, and 51.5% in HTN. However, in the GDM group, the highest frequency of T2DM was found in fathers (46.2%). The overall analysis confirms that a family history in mothers highly influences the development of GDM in pregnant women, although healthy women may also be prone to developing diabetes in the future because of a genetic predisposition. In conclusion, a family history of maternal inheritance is highly influential in Saudi women.

In this study, 95.6% of the women were on a normal diet during pregnancy and 4.4% of the women with GDM were receiving insulin, indicating that these women had elevated glucose levels during pregnancy. The mean FPG was 7.23 ± 0.85, PPBG was 9.90 ± 5.24, OGTT-1h was 13.65 ± 2.38, OGTT-2h was 11.98 ± 1.01, and HbA1c was 5.73 ± 0.39. All four patients had elevated glucose levels, and clinicians recommended insulin to control the glucose levels. Elevated glucose levels can increase macrosomia, and respiratory distress syndrome can occur in infants exposed to elevated glucose levels.

Our study had both limitations and strengths. A limitation of this study was the lack of documentation on hypovitaminosis. A strength of this study was the validation of the results via Sanger sequencing analysis. Another limitation was the small sample size. Measurement of the serum levels of 25-hydroxyvitamin D or vitamin D was another strength of this study. The final strength of this study was the enrollment of all pregnant Saudi women, which decreased study bias and will strengthen the effects of these data on policies. The final limitation of this study was the lack of certain metrics, including body composition, waist-to-hip circumference, sun exposure, physical activity, and post-pregnancy BMI details.

## 5. Conclusions

In conclusion, the ApaI, BsmI, and TaqI SNPs are associated with alleles, genotypes, and specific models for studying women with GDM in Saudi Arabia. The vitamin D levels were low in women with GDM and were not affected by the BMI of pregnant women. The data also revealed a positive association between different glucose parameters, including vitamin D levels in women with GDM. Further studies should be conducted with larger sample sizes, in which gene–gene and gene–environmental interactions are considered. Furthermore, these should use additional statistical analyses, such as linear regression, ANOVA, haplotype, and LD analysis, of specific ethnic populations to rule out whether they have any role associated with the *VDR* gene. Vitamin D levels are also important for understanding its role in the *VDR* gene, as well as in GDM.

## Figures and Tables

**Table 1:** SNP details of the *VDR* gene present in this study.

SNP	rs Number	Region	Position	Variant	Forward Primer	Reverse Primer	T_m_	PCR	Restriction Enzyme
ApaI	rs79785232	Intron-8	11740553	A > C	AGAGCATGGACAGGGAGC	GCAACTCCTCATGGCTGAGGTC	68 °C	746 bp	ApaI [GGGCC^↑^C]
BsmI	rs1544410	Intron-8	154441349	A > G	CAACCAAGACTACAACCG	AACCAGCGGAAGAGGTCAAGG	66 °C	872 bp	BsmI [G^↑^CATTC]
FokI	rs2228570	Exon-2	154441452	C > T	AGAGCATGGACAGGGAGC	GCAACTCCTCATGGCTGAGGTC	68 °C	267 bp	FokI [CGATG(N)^9↑^]
TaqI	rs731236	Exon-9	1540309	T > C	AGCTGGCCCTGGCACTGAC	ATGGAAACACCTTGCTTCTTCT	68 °C	746 bp	TaqI [T^↑^CGA]

**Table 2:** Comparison of the demographic characteristics’ information between GDM and non-GDM women.

Pregnant Women Parameters	GDM (*n* = 90)	Non-GDM (*n* = 90)	*p*-Values
Age (Years)	32.34 ± 5.42	28.26 ± 6.39	0.0001
Weight (kgs)	76.75 ± 12.85	73.57 ± 12.38	0.092
Height (cm)	158.03 ± 5.84	157.86 ± 5.11	0.835
BMI (kg/m^2^)	30.60 ± 4.87	29.39 ± 4.38	0.081
SBP (mmHg)	130.20 ± 11.95	120.77 ± 6.10	<0.0001
DBP (mmHg)	81.47 ± 7.84	78.00 ± 5.02	0.0005
FPG (mmol/L)	5.54 ± 1.69	4.55 ± 0.62	0.0004
PPBG (mmol/L)	8.51 ± 17.63	4.79 ± 0.83	<0.0001
OGTT-1h (mmol/L)	11.45 ± 1.42	7.05 ± 1.46	<0.0001
OGTT-2h (mmol/L)	9.81 ± 1.58	6.31 ± 1.54	<0.0001
HbA1c (%)	5.59 ± 0.52	5.26 ± 0.33	0.0008
TG (mmol/L)	1.87 ± 1.07	1.54 ± 2.11	0.187
TC (mmol/L)	5.39 ± 1.14	5.70 ± 1.27	0.086
HDLc (mmol/L)	0.71 ± 0.36	0.92 ± 0.44	0.0005
LDLc (mmol/L)	3.83 ± 0.86	3.71 ± 0.98	0.383
25 hydroxyvitamin-D (nmol/L)	47.83 ± 21.23	50.51 ± 20.59	0.391
R_x_ (Diet: Insulin)	86 (95.6%): 04 (4.4%)	NA	NA
Family History of GDM	23 (25.6%)	20 (22.2%)	0.087
Family History of T2DM	46 (51.1%)	39 (43.3%)	0.042
Family History of HTN	34 (37.8%)	33 (36.7%)	0.966

NA = Not applicable to this study.

**Table 3:** Allele frequencies calculated between GDM and non-GDM subjects.

rs Number	Alleles	GDM (*n* = 90)	Non-GDM (*n* = 90)	OR (95%CI) and *p*-Value
ApaI (rs79785232)	A	116 (64.4%)	131 (72.8%)	Reference
	C	64 (35.6%)	49 (27.2%)	OR-1.47 (95%CI: 0.94–42.31) *p* = 0.088
BsmI (rs1544410)	A	102 (71.1%)	132 (73.3%)	Reference
	G	78 (28.9%)	48 (26.7%)	OR-2.10 (95%CI: 1.35–3.24) *p* = 0.0009
FokI (rs2228570)	C	143 (79.4%)	146 (81.1%)	Reference
	T	37 (20.6%)	34 (18.9%)	OR-1.11 (95%CI: 0.66–1.86) *p* = 0.691
TaqI (rs731236)	T	122 (67.8%)	143 (79.4%)	Reference
	C	58 (32.2%)	37 (20.6%)	OR-1.83 (95%CI: 1.13–2.96) *p* = 0.012

**Table 4:** Statistical calculations obtained between GDM and non-GDM cases from four different RS number genotypes in the *VDR* gene.

Specific rs Number	Genotypes	GDM (*n* = 90)	Non-GDM (*n* = 90)	OR (95%CI) and *p*-Value
ApaI (rs79785232)	AA (Wild type)	35 (38.9%)	49 (54.4%)	Reference
	AC (Heterozygous co-dominant)	46 (51.1%)	33 (36.7%)	OR-1.95 (95%CI: 1.04–3.63) *p* = 0.034
	CC (Homozygous co-dominant)	09 (10%)	08 (8.9%)	OR-1.47 (95%CI: 0.51–4.22) *p* = 0.463
	AC + CC vs. AA (Dominant)	55 (61.1%)	41 (45.6%)	OR-1.87 (95%CI: 1.03–3.39) *p* = 0.036
	AA + CC vs. AC (Co-Dominant model)	44 (48.9%)	57 (63.3%)	OR-0.55 (95%CI: 0.30–1.01) *p* = 0.050
	CC vs. AC + AA (Recessive)	09 (10%)	08 (8.9%)	OR-1.13 (95%CI: 0.41–3.09) *p* = 0.798
	HWE analysis	ꭓ^2^ = 1.19; *p* = 0.27	ꭓ^2^ = 0.03; *p* = 0.85	
BsmI (rs1544410)	AA (Wild type)	31 (34.4%)	46 (51.1%)	Reference
	AG (Heterozygous co-dominant)	40 (44.5%)	40 (44.5%)	OR-1.48 (95%CI: 0.78–2.79) *p* = 0.220
	GG (Homozygous co-dominant)	19 (21.1%)	04 (4.4%)	OR-7.04 (95%CI: 2.18–22.72) *p* = 0.0003
	AG + GG vs. AA (Dominant)	59 (65.6%)	44 (48.9%)	OR-1.99 (95%CI: 1.09–3.62) *p* = 0.023
	AA + GG vs. AG (Co-dominant model)	50 (55.6%)	50 (55.6%)	OR-1.00 (95%CI: 0.55–1.80) *p* = 0.999
	GG vs. AG + AA (Recessive)	19 (21.1%)	4 (4.4%)	OR-5.75 (95%CI: 1.87–17.69) *p* = 0.0008
	HWE analysis	ꭓ^2^ = 0.81; *p* = 0.36	ꭓ^2^ = 1.67; *p* = 0.19	
FokI (rs2228570)	CC (Wild type)	59 (65.5%)	61 (67.8%)	Reference
	CT (Heterozygous co-dominant)	25 (27.8%)	24 (26.7%)	OR-1.07 (95%CI: 0.55–2.09) *p* = 0.826
	TT (Homozygous co-dominant)	06 (6.7%)	05 (5.5%)	OR-1.24 (95%CI: 0.35–4.28) *p* = 0.099
	TC + CC vs. TT (Dominant model)	31 (34.4%)	29 (32.2%)	OR-1.10 (95%CI: 0.59–2.05) *p* = 0.751
	CC + TT vs. CT (Co-dominant model)	65 (72.2%)	66 (73.3%)	OR-0.94 (95%CI: 0.49–1.82) *p* = 0.867
	CC vs. TC + TT (Recessive model)	19 (21.1%)	11 (12.2%)	OR-1.92 (95%CI: 0.85–4.31) *p* = 0.109
	HWE analysis	ꭓ^2^ = 2.01; *p* = 0.15	ꭓ^2^ = 1.51; *p* = 0.21	
TaqI (rs731236)	TT (Wild type)	43 (47.8%)	57 (63.3%)	Reference
	TC (Heterozygous co-dominant)	36 (40.0%)	29 (32.2%)	OR-1.64 (95%CI: 0.87–3.08) *p* = 0.119
	CC (Homozygous mutant)	11 (12.2%)	04 (4.5%)	OR-3.64 (95%CI: 1.08–12.23) *p* = 0.028
	TC + CC vs. TT (Dominant model)	47 (52.2%)	33 (36.7%)	OR-1.88 (95%CI: 1.04–3.42) *p* = 0.035
	CC + TT vs. TC (Co-dominant model)	54 (60%)	61 (67.8%)	OR-0.71 (95%CI: 0.38–1.31) *p* = 0.278
	CC vs. TC + TT (Recessive model)	11 (12.2%)	04 (4.5%)	OR-2.99 (95%CI: 0.91–9.78) *p* = 0.059
	HWE analysis	ꭓ^2^ = 0.63; *p* = 0.42	ꭓ^2^ = 0.01; *p* = 0.89	

**Table 5:** Differentiation of vitamin D values, sub-categorized according to the BMI levels in GDM and non-GDM women.

Pregnant Women	Vitamin D (Categories)				Total	ꭓ2	*p*-Value
	Deficient (<30 nmol/L)	Insufficient (30–50 nmol/L)		Sufficient (>50 nmol/L)			
GDM (*n* = 90)	24 (26.7%)	28 (31.1%)		38 (42.2%)	90 (100%)		
Non-GDM (*n* = 90)	17 (18.9%)	25 (27.8%)		48 (53.3%)	90 (100%)	2.528	0.283
Total (*n* = 180)	41 (22.8%)	53 (29.4%)		86 (47.8%)	180 (100%)		
**Pregnant Women**	**BMI (Categories)**				**Total**	**ꭓ2**	***p*-Value**
	**Normal BMI**	**Overweight**	**Obese**	**Morbidly Obese**			
GDM (*n* = 90)	13 (14.4%)	25 (27.8%)	37 (41.1%)	15 (16.7%)	90 (100%)		
Non-GDM (*n* = 90)	15 (16.7%)	31 (34.4%)	38 (42.2%)	06 (6.7%)	90 (100%)	4.656	0.199
Total (*n* = 180)	28 (15.6%)	56 (31.1%)	75 (41.7%)	21 (11.7%)	180 (100%)		

**Table 6:** Comparison of the vitamin D serum levels and GDM covariates using ANOVA analysis.

Covariates	Deficient Levels: <30 nmol/L (*n* = 24)	Insufficient Levels: 30–50 nmol/L (*n* = 28)	Sufficient Levels: >50 nmol/L (*n* = 38)	*p*-Value
Age (years)	33.42 ± 5.06	30.13 ± 4.80	29.21 ± 4.00	0.402
Weight (kgs)	76.62 ± 13.95	75.99 ± 13.32	77.39 ± 12.07	0.722
BMI (kg/m^2^)	30.64 ± 4.89	29.96 ± 4.89	31.05 ± 4.92	0.992
SBP (mmHg)	130.42 ± 12.07	129.18 ± 11.62	130.82 ± 12.38	0.940
DBP (mmHg)	82.79 ± 7.92	79.75 ± 7.61	81.89 ± 7.95	0.967
FPG (mmol/L)	5.30 ± 2.08	5.30 ± 0.71	5.87 ± 1.91	0.0001
PPBG (mmol/L)	6.70 ± 2.53	6.90 ± 2.80	10.81 ± 26.99	<0.0001
OGTT-1h (mmol/L)	11.20 ± 0.91	11.50 ± 1.33	11.58 ± 1.73	0.005
OGTT-2h (mmol/L)	9.48 ± 1.36	9.81 ± 1.27	10.01 ±1.88	0.060
HbA1c (%)	5.58 ± 0.56	5.65 ± 0.49	5.55 ± 0.52	0.802
TG (mmol/L)	1.77 ± 1.16	2.13 ± 1.15	1.74 ± 0.93	0.385
TC (mmol/L)	5.70 ± 1.23	5.41 ± 0.97	5.17 ± 1.19	0.435
HDLc (mmol/L)	0.77 ± 0.43	0.70 ± 0.41	0.66 ± 0.27	0.022
LDLc (mmol/L)	4.09 ± 0.97	3.79 ± 0.50	3.70 ± 0.98	0.001

**Table 7:** Correlation between the vitamin D levels and SNPs present in the *VDR* gene in GDM women.

	ApaI AA Genotypes (*n* = 35)	ApaI AC Genotypes (*n* = 46)	ApaI CC Genotypes (*n* = 09)	*p*-Value
Deficient levels (<30 nmol/L)	24.0 ± 1.22	23.43 ± 3.41	24.0 ± 2.64	0.601
Insufficient levels (30–50 nmol/L)	39.01 ± 5.92	38.64 ± 4.48	37.67 ± 4.04	0.775
Sufficient levels (>50 nmol/L)	67.26 ± 11.51	71.93 ± 10.94	72.00 ± 17.43	<0.0001
	**BsmI AA Genotypes (*n* = 31)**	**BsmI AG Genotypes (*n* = 40)**	**BsmI GG Genotypes (*n* = 19)**	
Deficient levels (<30 nmol/L)	23.55 ± 2.61	23.37 ± 3.29	24.00 ± 3.26	0.762
Insufficient levels (30–50 nmol/L)	38.22 ± 4.73	38.67 ± 4.87	42.25 ± 5.73	0.016
Sufficient levels (>50 nmol/L)	71.15 ± 11.83	68.94 ± 11.19	68.50 ± 13.43	0.667
	**FokI CC Genotypes (*n* = 59)**	**FokI CT Genotypes (*n* = 25)**	**FokI TT Genotypes (*n* = 06)**	
Deficient levels (<30 nmol/L)	23.35 ± 2.97	23.75 ± 3.10	25.00 ± 2.82	0.415
Insufficient levels (30–50 nmol/L)	39.56 ± 5.12	39.60 ± 4.19	32.00 ± 1.41	0.001
Sufficient levels (>50 nmol/L)	70.48 ± 11.92	67.57 ± 11.75	64.00 ± 8.48	0.309
	**TaqI TT Genotypes (*n* = 43)**	**TaqI TC Genotypes (*n* = 36)**	**TaqI CC Genotypes (*n* = 11)**	
Deficient levels (<30 nmol/L)	23.83 ± 2.75	23.00 ± 3.26	25.50 ± 2.12	0.045
Insufficient levels (30–50 nmol/L)	37.75 ± 4.31	38.63 ± 5.55	43.00 ± 3.46	0.152
Sufficient levels (>50 nmol/L)	71.52 ± 12.21	66.93 ± 11.46	70.50 ± 9.88	0.216

**Table 8:** Multiple linear regression model of the SNPs presents in the *VDR* gene in GDM women.

Covariates	R-Value ^a^	Adjusted R Square Value	Standardized β-Coefficient for rs79785232	Standardized β-Coefficient for rs1544410	Standardized β-Coefficient for rs2228570	Standardized β-Coefficient for rs731236	F	*p*-Value ^b^
Age	0.188	−0.010	0.117	−0.025	0.106	−0.084	0.775	0.544
Weight	0.175	−0.015	−0.173	0.007	0.032	0.009	0.672	0.613
BMI	0.205	−0.003	−0.190	−0.078	0.088	−0.036	0.936	0.447
SBP	0.142	−0.026	0.009	−0.014	−0.085	0.134	0.439	0.780
DBP	0.117	−0.033	0.027	0.037	−0.109	0.029	0.294	0.881
FPG	0.195	−0.007	0.152	−0.068	−0.060	−0.054	0.840	0.504
PPBG	0.154	−0.022	−0.121	−0.022	−0.036	−0.089	0.520	0.722
OGTT-1h	0.400	0.120	−0.158	−0.103	0.372	−0.066	4.041	0.005
OGTT-2h	0.234	0.010	−0.076	−0.110	0.196	−0.058	1.230	0.304
HbA1c	0.198	−0.006	0.094	0.181	0.024	−0.070	0.871	0.485
TC	0.190	−0.009	0.096	0.055	−0.162	−0.007	0.797	0.531
TG	0.187	−0.010	0.142	0.034	−0.128	−0.020	0.770	0.548
HDLc	0.108	−0.035	−0.023	0.018	0.084	0.050	0.250	0.909
LDLc	0.281	0.036	0.112	−0.049	−0.242	−0.068	1.825	0.131
25 hydroxyvitamin-D	0.248	0.018	−0.150	−0.067	−0.190	0.013	1.398	0.242

^a^ indicates predictors (constants) and ^b^ indicates dependent variables as listed in covariates.

**Table 9:** One-way ANOVA of the genotypes present in the VDR–SNPs and GDM covariates.

	ApaI (rs79785232)				BsmI (rs1544410)				FokI (rs2228570)				TaqI (rs731236)			
	AA (*n* = 35)	AC (*n* = 46)	CC (*n* = 09)	TT (*n* = 43)	TT (*n* = 43)	TT (*n* = 43)	TT (*n* = 43)	*p*-Value	CC (*n* = 59)	CT (*n* = 25)	TT (*n*=06)	*p*-Value	TT (*n* = 43)	TC (*n* = 36)	CC (*n* = 11)	*p*-Value
Age	31.94 ± 5.64	32.02 ± 5.39	35.56 ± 3.88	32.93 ± 5.51	32.93 ± 5.51	32.93 ± 5.51	32.93 ± 5.51	0.255	31.95 ± 5.17	32.96 ± 5.00	33.67 ± 9.31	0.095	32.93 ± 5.51	31.64 ± 5.34	32.36 ± 5.57	0.976
Weight	79.08 ± 12.96	75.94 ± 12.97	71.84 ± 11.04	77.33 ± 12.27	77.33 ± 12.27	77.33 ± 12.27	77.33 ± 12.27	0.139	76.20 ± 12.87	78.95 ± 13.29	73.04 ± 11.13	0.890	77.33 ± 12.27	74.71 ± 12.24	81.16 ± 16.56	0.401
BMI	31.56 ± 5.01	30.13 ± 4.76	29.28 ± 4.96	30.96 ± 4.46	30.96 ± 4.46	30.96 ± 4.46	30.96 ± 4.46	0.822	30.32 ± 4.73	31.16 ± 4.64	31.02 ± 7.48	0.273	30.96 ± 4.46	29.83 ± 4.65	31.75 ± 6.90	0.151
SBP	130.57 ± 12.64	129.76 ± 11.20	131.01 ± 14.20	128.44 ± 11.75	128.44 ± 11.75	128.44 ± 11.75	128.44 ± 11.75	0.941	130.92 ± 12.30	128.32 ± 11.21	131.01 ± 12.57	0.863	128.44 ± 11.75	132.03 ± 12.58	131.09 ± 10.55	0.782
DBP	82.09 ± 7.31	80.46 ± 8.05	84.22 ± 8.73	81.05 ± 7.90	81.05 ± 7.90	81.05 ± 7.90	81.05 ± 7.90	0.922	82.05 ± 8.01	80.52 ± 7.58	79.67 ± 7.94	0.952	81.05 ± 7.90	82.36 ± 7.77	80.18 ± 8.30	0.966
FPG	5.29 ± 0.89	5.58 ± 1.75	6.30 ± 3.22	5.63 ± 2.22	5.63 ± 2.22	5.63 ± 2.22	5.63 ± 2.22	0.030	5.64 ± 2.00	5.23 ± 0.67	5.83 ± 1.43	0.0001	5.63 ± 2.22	5.56 ± 0.97	5.12 ± 1.12	0.0002
PPBG	11.27 ± 28.10	6.77 ± 2.79	6.58 ± 1.79	10.38 ± 25.36	10.38 ± 25.36	10.38 ± 25.36	10.38 ± 25.36	0.0001	9.39 ± 21.70	6.73 ± 2.68	7.12 ± 2.29	0.0005	10.38 ± 25.36	6.65 ± 2.70	7.21 ± 3.16	0.00001
OGTT-1h	11.51 ± 1.18	11.53 ± 1.62	10.83 ± 1.19	11.40 ± 1.41	11.40 ± 1.41	11.40 ± 1.41	11.40 ± 1.41	0.068	11.11 ± 1.21	11.96 ± 1.34	12.72 ± 2.41	0.045	11.40 ± 1.41	11.56 ± 1.31	11.32 ± 1.90	0.296
OGTT-2h	9.84 ± 1.71	9.81 ± 1.62	9.63 ± 0.75	9.81 ± 1.61	9.81 ± 1.61	9.81 ± 1.61	9.81 ± 1.61	0.332	9.59 ± 1.51	10.18 ± 1.60	10.40 ± 1.99	0.664	9.81 ± 1.61	9.86 ± 1.60	9.62 ± 1.21	0.549
HbA1c	5.53 ± 0.54	5.63 ± 0.52	5.59 ± 0.47	5.61 ± 0.56	5.61 ± 0.56	5.61 ± 0.56	5.61 ± 0.56	0.797	5.58 ± 0.49	5.64 ± 0.61	5.47 ± 0.42	0.355	5.61 ± 0.56	5.58 ± 0.49	5.51 ± 0.47	0.641
TG	1.89 ± 1.11	1.74 ± 1.01	2.44 ± 1.14	1.88 ± 1.08	1.88 ± 1.08	1.88 ± 1.08	1.88 ± 1.08	0.455	1.98 ± 1.15	1.73 ± 0.96	1.36 ± 0.33	0.017	1.88 ± 1.08	1.91 ± 1.16	1.68 ± 0.70	0.204
TC	5.31 ± 0.90	5.33 ± 1.32	5.99 ± 0.93	5.36 ± 1.00	5.36 ± 1.00	5.36 ± 1.00	5.36 ± 1.00	0.839	5.49 ± 1.28	5.20 ± 0.80	5.13 ± 0.89	0.034	5.36 ± 1.00	5.55 ± 1.29	4.95 ± 1.15	0.296
HDLc	0.69 ± 0.40	0.74 ± 0.35	0.58 ± 0.23	0.68 ± 0.35	0.68 ± 0.35	0.68 ± 0.35	0.68 ± 0.35	0.366	0.69 ± 0.40	0.69 ± 0.28	0.88 ± 0.07	0.0005	0.68 ± 0.35	0.71 ± 0.31	0.77 ± 0.54	0.056
LDLc	3.81 ± 0.65	3.76 ± 0.98	4.29 ± 0.90	3.87 ± 0.79	3.87 ± 0.79	3.87 ± 0.79	3.87 ± 0.79	0.419	3.98 ± 0.95	3.59 ± 0.62	3.41 ± 0.47	0.022	3.87 ± 0.79	3.93 ± 0.88	3.38 ± 1.01	0.561
Vitamin D	52.49 ± 19.32	44.93 ± 22.03	44.56 ± 23.25	48.79 ± 22.75	48.79 ± 22.75	48.79 ± 22.75	48.79 ± 22.75	0.814	50.92 ± 22.09	42.36 ± 18.65	40.33 ± 19.03	0.611	48.79 ± 22.75	46.08 ± 20.50	49.82 ± 18.63	0.675

**Table 10:** Haplotype analysis of the SNPs present in the VDR gene for GDM cases.

S. No	rs79785232	rs1544410	rs2228570	rs731236	Freq	OR (95%CI)	*p*-Value
1	A	A	C	T	0.2556	1.00	-
2	A	G	C	T	0.1799	1.59 (0.62–4.07)	0.33
3	C	A	C	T	0.1534	1.51 (0.67–3.41)	0.32
4	A	A	T	C	0.0674	0.97 (0.29–3.17)	0.95
5	A	G	C	C	0.0612	5.30 (1.34–21.01)	0.019
6	A	A	C	C	0.0558	1.81 (0.31–10.55)	0.51
7	C	G	C	T	0.0529	3.62 (0.92–14.17)	0.067
8	A	A	T	T	0.0441	0.59 (0.08–4.51)	0.61
9	C	A	C	C	0.0341	3.30 (0.48–22.83)	0.23
10	C	A	T	T	0.0294	6.91(0.78–32.19)	<0.0001
11	C	G	T	C	0.0145	12.1(3.67–46.9)	<0.0001
12	A	G	T	T	0.0114	5.38 (0.20–146.61)	0.32
13	A	G	T	C	0.0107	2.86 (0.81–16.75)	<0.0001
14	C	A	T	C	0.0103	0.47 (0.29–2.17)	0.86
15	C	G	T	T	0.0095	1.29 (0.62–3.07)	0.32
16	C	A	T	T	0.0099	4.30 (0.88–29.83)	<0.0001

**Table 11:** Analysis of linkage disequilibrium for SNPs in the VDR gene in pregnant women.

Pregnant Women	L1	L2	D’	r^2^
GDM women	rs731236	rs2228570	0.256	0.036
GDM women	rs731236	rs1544410	0.068	0.003
GDM women	rs731236	rs79785232	0.159	0.007
GDM women	rs2228570	rs1544410	0.231	0.011
GDM women	rs2228570	rs79785232	0.174	0.014
GDM women	rs1544410	rs79785232	0.221	0.021
Non-GDM women	rs731236	rs2228570	0.414	0.154
Non-GDM women	rs731236	rs1544410	0.509	0.024
Non-GDM women	rs731236	rs79785232	0.232	0.005
Non-GDM women	rs2228570	rs1544410	0.581	0.029
Non-GDM women	rs2228570	rs79785232	0.062	0.001
Non-GDM women	rs1544410	rs79785232	0.388	0.021

**Table 12:** Gene-gene interaction analysis estimating the risk of GDM.

Model No.	Best Combination of Genes	Training Accuracy	Testing Accuracy	CVC	*p*-Value	Total Sensitivity	Total Specificity	ꭓ^2^	OR (95%CI)	F-Measure	Kappa
1	rs1544410	0.5965	0.5001	7/10	0.015	0.6629	0.5165	5.91	2.10 (1.15–3.83)	0.614	0.179
2	rs1544410, rs731236	0.6336	0.5544	7/10	0.002	0.4719	0.7912	13.90	3.38 (1.75–6.51)	0.56	0.264
3	rs1544410, rs2228570, rs731236	0.6658	0.5714	9/10	<0.001	0.573	0.7473	19.06	3.96 (2.10–7.46)	0.625	0.321
4	rs79785232, rs1544410, rs2228570, rs731236	0.6881	0.5951	10/10	<0.001	0.7416	0.6264	24.67	4.81 (2.54–9.09)	0.698	0.367

## Data Availability

Not applicable.
